# An Expedient Regio- and Diastereoselective Synthesis of Hybrid Frameworks with Embedded Spiro[9,10]dihydroanthracene [9,3′]-pyrrolidine and Spiro[oxindole-3,2′-pyrrolidine] Motifs via an Ionic Liquid-Mediated Multicomponent Reaction

**DOI:** 10.3390/molecules200916142

**Published:** 2015-09-03

**Authors:** Natarajan Arumugam, Abdulrahman I. Almansour, Raju Suresh Kumar, J. Carlos Menéndez, Mujeeb A. Sultan, Usama Karama, Hazem A. Ghabbour, Hoong-Kun Fun

**Affiliations:** 1 Department of Chemistry, College of Science, King Saud University, P. O. Box 2455, Riyadh 11451, Saudi Arabia; E-Mails: almansor@ksu.edu.sa (A.I.A.); sraju@ksu.edu.sa (R.S.K.); alhosami1983@yahoo.co.uk (M.A.S.); karama@ksu.edu.sa (U.K.); 2 Departamento de Química Orgánica y Farmacéutica, Facultad de Farmacia, Universidad Complutense, Madrid 28040, Spain; 3 Department of Pharmaceutical Chemistry, College of Pharmacy, King Saud University, P.O. Box 2457, Riyadh 11451, Saudi Arabia; E-Mails: ghabbourh@yahoo.com (H.A.G.); hfun.c@ksu.edu.sa (H.-K.F.); 4 X-Ray Crystallography Unit, School of Physics, Universiti Sains Malaysia, Penang 11800, Malaysia

**Keywords:** multicomponent reactions, 1,3-dipolar cycloaddition reactions, spirooxindoles, spiropyrrolidines, ionic liquids

## Abstract

A series of hitherto unreported anthracene-embedded dispirooxindoles has been synthesized via a one-pot three-component 1,3-dipolar cycloaddition reaction of an azomethine ylide, generated *in situ* from the reaction of isatin and sarcosine to 10-benzylideneanthracen-9(10*H*)-one as a dipolarophile in 1-butyl-3-methylimidazolium bromide([bmim]Br), an ionic liquid. This reaction proceeded regio- and diastereoselectively, in good to excellent yields.

## 1. Introduction

The creation of molecular complexity and diversity in potential drug candidates and biologically important molecules from common starting materials while combining favorable economic and environmental aspects constitutes a great challenge in modern organic chemistry from both academic and industrial perspectives [1,2]. One protocol to realize these goals involves the use of multi-component reactions (MCRs), which enable the creation of several bonds in a single operation and offer remarkable advantages such as convergence, operational simplicity, facile automation, reduction in the number of workup steps and minimization of extraction and purification processes and waste generation, rendering the transformations green. MCRs, besides facilitating the expedient creation of chemical libraries of structurally diverse drug-like compounds [3,4,5], play a key role in combinatorial synthesis [6,7] and, generally speaking, in drug discovery [8,9,10].

Another important aspect of green chemistry pertains to the elimination of volatile organic solvents or their replacement by non-inflammable, non-volatile, non-toxic and inexpensive green solvents. In this context, ionic liquids are widely recognized as green solvents in organic synthesis because of their unique properties such as high chemical and thermal stability, solvating ability, behavior as acidic and basic catalysts and recyclability. For this reason, their use in organic synthesis has emerged as an important facet of green chemistry. Interestingly, the solubility, density, refractive index, viscosity, acidic or basic character and associated catalyzing ability of ionic liquids can be tuned by judicious modification of the structure of their anion/cation to suit different applications. Consequently, ionic liquids are also referred to as ‘designer solvents’, although this term is perhaps too narrow [11,12]. Owing to these green credentials, ionic liquids have attracted great interest as environmentally benign reaction media [13], catalysts [14] and reagents [15,16], besides having many other applications. In particular, the ionic liquid, 1-butyl-3-methylimidazolium bromide ([bmim]Br) has gained importance in organic synthesis; however, it has been less investigated in literature. There is much interest in the burgeoning field of the combination of multiple bond-forming reactions with the use of ionic liquids as reaction media as two mutually reinforcing strategies towards sustainable synthesis. Thus, the use of ionic liquids as reaction media for multicomponent reactions [17,18,19,20,21] and cycloaddition reactions (including 1,3-dipolar cycloadditions [22,23]) are particularly interesting, if relatively little explored, areas.

**Figure 1:**
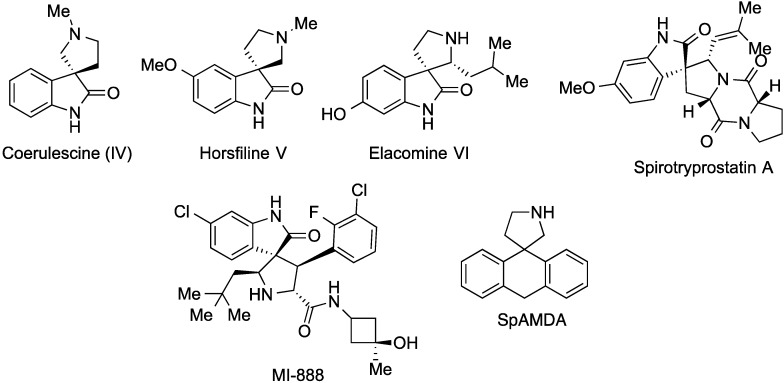
Some biologically relevant derivatives of the spiro[9,10]dihydroanthracene [9,3′]-pyrrolidine and spiro[oxindole-3,2′-pyrrolidine] frameworks.

Spiropyrrolidine-oxindole scaffolds are embodied in many alkaloids such as horsfiline, elacomine and coerulescine, which are inhibitors of the mammalian cell cycle at the G2/M interphase [24,25], More complex natural spirooxindoles *viz*. the spirotryprostatins, also showed anticancer activity [26]. More importantly, some synthetic spirooxindoles such as MI-888 have been in preclinical research for the treatment of human cancers [27]. On the other hand, spiro[9,10-dihydroanthracene]-9,3′-pyrrolidine (SpAMDA) is a high-affinity antagonist of the 5-HT2A receptor (Figure 1) [28,29]. Such antagonists are important coadjuvants in cancer chemotherapy; thus, levomepromazine, primarily acting as a 5HT-2 antagonist at the vomiting center, is useful as a broad-spectrum agent for resistant nausea in cancer chemotherapy, especially in children [30].

**Figure 2:**
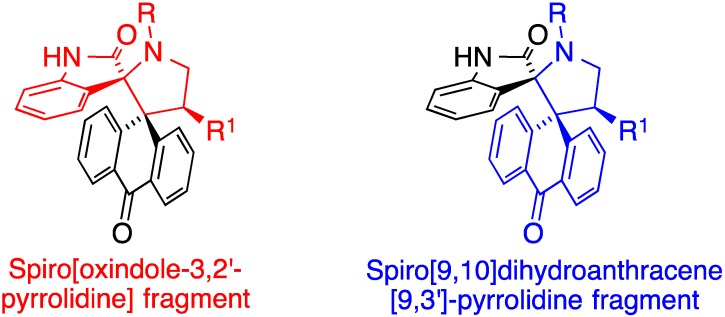
Hybrid structure of the compounds targeted in this paper.

Based on the precedents outlined above, we reasoned that the combination of the spiro[9,10]dihydroanthracene [9,3′]-pyrrolidine and spiro[oxindole-3,2′-pyrrolidine] motifs in a single molecule, as shown in Figure 2, would be of interest in the context of anticancer drug discovery. In continuation of our interest in the area of 1,3-dipolar cycloaddition reactions [31,32,33,34,35,36], we describe in this article the preparation of these compounds by application of a three-component process having as the key step a 1,3-dipolar cycloaddition of an azomethine ylide to an olefinic dipolarophile [37,38,39,40]. These reactions were performed in ([bmim]Br), an ionic liquid that has been previously employed as the reaction medium for this kind of chemistry [23,41], although it has received relatively little attention in comparison to others.

## 2. Results and Discussion

A series of 10-benzylideneanthracen-9(10*H*)-ones **3a**–**k** were prepared by the acid-catalyzed condensation of anthracen-9(10*H*)-one **1** with substituted benzaldehydes **2a**–**2k**, following a literature procedure [41]. The three-component reactions of compounds **3** with non-stabilized azomethine ylide **8**, generated *in situ* by the decarboxylative condensation of isatin **4** and an α-amino acid, sarcosine **5** was first studied in terms of solvent optimization for the model three-component reaction between 10-benzylideneanthracen-9(10*H*)-one (**3h**; 1 mmol), isatin (**4**; 1 mmol) and sarcosine (**5**; 1 mmol). The reaction failed in ethanol, methanol, dioxane, and a dioxane/methanol (1:1 *v*/*v*) mixture under reflux conditions. As shown in Table 1, the same starting materials were heated in DMF at several temperatures (entries 1–3), and the desired product **6h** was obtained in 65% yield at 100 °C (entry 3). Finally, the reaction was carried out in presence of an ionic liquid, 1-butyl-3-methylimidazolium bromide ([bmim]Br at 100 °C to furnish **6h** in an excellent yield (89%) and in a short reaction time compared to DMF (entry 4). We also verified that the ionic liquid could be used three times without any significant loss in yield (entries 5–7).

**Table 1:** Solvent-screen for the synthesis of heterocyclic hybrid **6h**. ^a^

Entry	Solvent	Temp, °C	Time, h	Yield, % ^b^
1	DMF	60	4	45
2	DMF	80	4	50
3	DMF	100	3	65
4	[BIMm]Br	100	2	89
5	[BIMm]Br	100	2	89
6	[BIMm]Br	100	2	87
7	[BIMm]Br	100	2	87

^a^ The reaction did not proceed in refluxing ethanol, methanol, dioxane and 1/1 dioxane/methanol. ^b^ Isolated yield after purification by column chromatography.

**Scheme 1:**
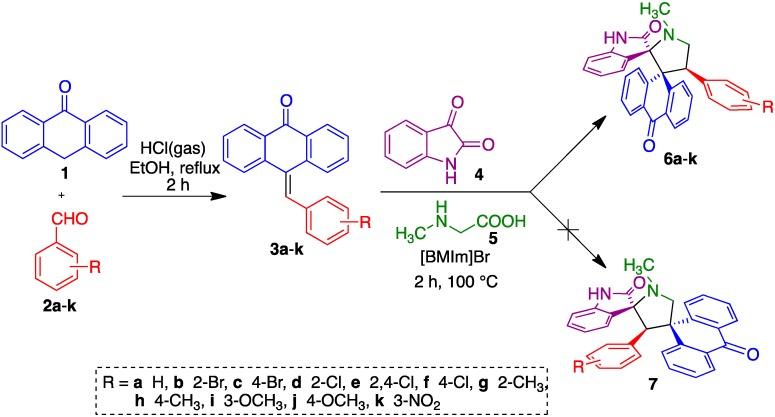
Synthesis of 9-arylmethylene-10-anthrone derivatives **3** and their transformation into the target *bi*spiro compounds **6**.

Following the optimization study, all subsequent reactions were effected by heating an equimolar mixture of the reactants in [bmim]Br (3 mL) in an oil bath at 100 °C for 2 h. After completion of the reaction (TLC), the product was isolated and purified through column chromatography to furnish the target compounds **6** in excellent yields, whilst the ionic liquid could be recovered and reused by simple drying under vacuum. No traces were observed of the other possible regioisomer of **6**, *i.e.*, structure **7** (Scheme 1). As shown by the examples summarized in Table 2, the aromatic substituent tolerated hydrogen, electron-releasing (Me, OMe) and electron-withdrawing (Cl, Br, NO_2_) substituents at its *ortho*, *meta* and *para* positions.

**Table 2:** Scope of the synthesis of compounds **6**.

Entry	Compound	R	Yield, % ^a^
1	**6a**	H	81
2	**6b**	2-Br	80
3	**6c**	4-Br	85
4	**6d**	2-Cl	87
5	**6e**	2,4-Cl_2_	84
6	**6f**	4-Cl	88
7	**6g**	2-Me	80
8	**6h**	4-Me	89
9	**6i**	3-OMe	81
10	**6j**	4-OMe	79
11	**6k**	3-NO_2_	77

^a^ Isolated yield after purification by column chromatography.

**Figure 3:**
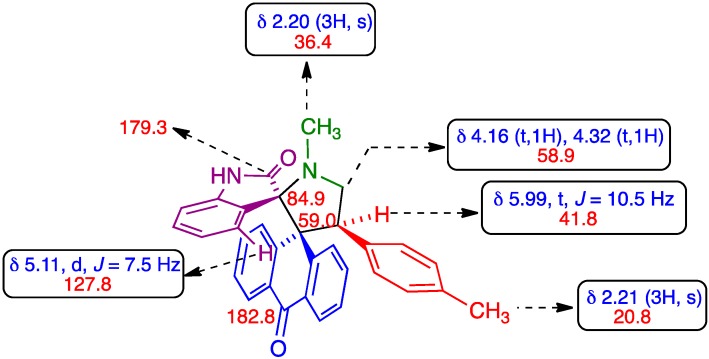
Key NMR data of model compound **6h**.

**Figure 4:**
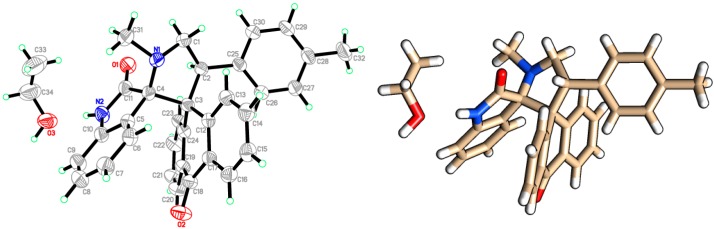
Two views of the X-Ray diffraction study of compound **6h**, crystallized with one molecule of ethanol.

The structure of compounds **6** was elucidated using ^1^H-, ^13^C- and 2D-NMR spectroscopic data as described below for **6h**. The ^1^H NMR spectrum of **6h** demonstrated a singlet at δ 2.20 ppm due to the *N*-methyl protons of pyrrolidine ring, which shows HMBCs with C-2 at δ 84.9 ppm and C-5 at δ 58.9 ppm (Figure 3). From C,H-correlations, the two triplets at δ 4.16 and 4.32 ppm were assigned to 5-CH_2_ protons. The benzylic proton (H-4) appeared as triplet at δ 5.99 ppm. The oxindole aromatic (C-4′) hydrogen unusually resonated at δ 5.11 ppm as doublet owing to the influence of the spatial proximity of one of the aromatic anthrone rings. The signals at δ 59.0 and 84.9 were assigned as anthrone and oxindole spirocarbons, respectively. The signals at 179.3 and 182.8 were due to the oxindole and anthrone carbonyl carbon, respectively. Unambiguous assignment of carbon C-2, C-3 C-4, C-5 of **6h** to the signals δ 84.9, 59.0, 41.8 and 58.9 ppm was made from their proton chemical shifts and their respective C, H-COSY correlations. The aromatic protons appear as multiplets in the range 6.37–8.00 ppm. The presence of a molecular ion peak at *m*/*z* = 472 (M^+^) in the mass spectrum confirms the formation of cycloadduct **6h** (*vide* Supplementary Data). Finally, the structure and stereochemistry of cycloadduct **6** was elucidated unambiguously by a single crystal X-Ray diffraction study of **6h** (Figure 4) [42].

**Scheme 2:**
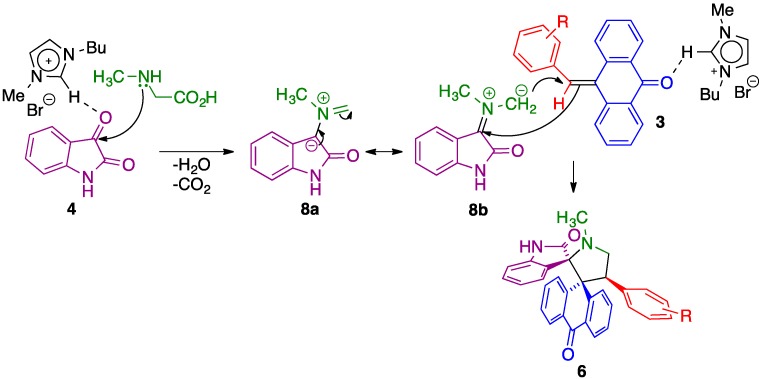
Mechanism proposed to account for the formation of compounds **6**.

A feasible mechanism proposed to rationalize for the formation of dispiropyrrolidines **6** is summarized in Scheme 2. Initially, the interaction of [bmim]Br with the carbonyl group of isatin via hydrogen bonding would increase the electrophilicity of the carbonyl carbon facilitating the nucleophilic attack of the NH of sarcosine. The subsequent dehydration and decarboxylation furnishes an azomethine ylide, which can be described by the **8a** and **8b** resonant forms. Similarly to the initial step, the interaction of [bmim]Br with the carbonyl group of 10-benzylideneanthracen-9(10*H*)-one presumably activates the exocyclic double bond, facilitating the addition of the azomethine ylide to the more electron deficient carbon of **3** to afford spiropyrrolidine **6**.

## 3. Experimental Section

Melting points were taken using open capillary tubes and are uncorrected. ^1^H, ^13^C and two-dimensional NMR spectra were recorded on a Varian Mercury JEOL-400 NMR spectrometer (Tokyo, Japan) and Bruker 300 MHz NMR spectrometers (Faellanden, Switzerland) in CDCl_3_ using TMS as internal standard. Standard Bruker software was used throughout. Chemical shifts are given in parts per million (δ-scale) and the coupling constants are given in Hertz. Single crystal X-Ray data set for **6h** was collected on Bruker APEXII D8 Venture diffractometer (Karlsruhe, Germany) with Mo Kα (λ = 0.71073 Å) radiation. Elemental analyses were performed on a Perkin Elmer 2400 Series II Elemental CHNS analyzer (Waltham, MA, USA).

General procedure for synthesis of dispirooxindolopyrrolidine fused anthrone **6a**–**k**: An equimolar mixture of 10-benzylideneanthracen-9(10*H*)-ones **3**, isatin **4** and sarcosine **5** were heated with stirring in [bmim]Br (3 mL) medium for 2 h at 100 °C. After completion of the reaction (TLC), the ethyl acetate (2 × 5 mL) was added and reaction mixture stirred 15 min. The ethyl acetate layer was separated, washed with water and dried. The products obtained in good yield were purified by column chromatography. The ionic liquid [bmim]Br, after extraction of the product, was completely dried under vacuum and reused for subsequent reactions.

(2′S*,4′S*)-1′-Methyl-4′-phenyl-10H-dispiro[anthracene-9,3′-pyrrolidine-2′,3′′-indoline]-2′′,10-dione (**6a**). White solid (262 mg, 81%); ^1^H-NMR (400 MHz, CDCl_3_): δ_H_ 2.22 (s, 3H, *N*CH_3_), 4.18 (t, *J* = 7.36 Hz, 1H), 4.35 (t, *J* = 8.8 Hz, 1H), 5.11 (d, *J* = 7.36 Hz, 1H), 6.03 (dd, *J* = 11, 7.32 Hz, 1H), 6.37–8.00 (m, Ar-H, 16H), 8.49 (s, 1H, N-H); ^13^C-NMR (100 MHz, CDCl_3_): δ 39.9, 42.1, 58.8, 59.0, 84.9, 108.9, 110.4, 113.6, 121.5, 125.6, 125.7, 126.6, 127.1, 127.2, 127.8, 127.9, 128.0, 128.1, 130.5, 131.4, 133.4, 135.5, 139.5, 140.0, 141.1, 141.8, 156.4, 178.6, 182.7. Mass spectrum (EI, 70 eV): *m*/*z*, 457 (M^+^). Anal. calcd. for C_31_H_24_N_2_O_2_: C, 81.56; H, 5.30; N, 6.14%. Found: C, 81.66; H, 5.47; N, 6.28%.

(2′S*,4′S*)-1′-Methyl-4′-(2-bromophenyl)-10H-dispiro[anthracene-9,3′-pyrrolidine-2′,3′′-indoline]-2′′,10-dione (**6b**). Pale yellow solid (237 mg, 80%); ^1^H-NMR (CDCl_3_, 300 MHz): δ 2.24 (s, 3H, *N*CH_3_), 4.21 (t, *J* = 7.36 Hz, 1H, *N*CH), 4.38 (t, *J* = 8.8 Hz, 1H, *N*CH), 5.10 (d, *J* = 7.36 Hz, 1H), 6.01 (dd, *J* = 11, 7.32 Hz, 1H), 6.20–7.92 (m, Ar-H, 15H), 8.39 (s, 1H,); ^13^C-NMR (CDCl_3_, 75 MHz): δ 39.8, 41.2, 58.4, 59.2, 84.4, 109.0, 110.3, 113.5, 121.0, 123.3, 125.4, 125.5, 124.6, 125.7, 125.8, 126.6, 127.1, 127.2, 127.3, 127.4, 127.6, 128.2, 130.9, 131.5, 132.8, 133.2, 138.7, 139.8, 140.8, 179.7, 182.5. Mass spectrum (EI, 70 eV): *m*/*z*, 536 (M^+^). Anal. calcd. for C_31_H_23_BrN_2_O_2_: C, 69.54; H, 4.33; N, 5.23; %. Found: C, 69.66; H, 4.45; N, 5.15%.

(2′S*,4′S*)-1′-Methyl-4′-(4-bromophenyl)-10H-dispiro[anthracene-9,3′-pyrrolidine-2′,3′′-indoline]-2′′,10-dione (**6c**). Pale yellow solid (252 mg, 85%); ^1^H-NMR (CDCl_3_, 400 MHz): δ 2.54 (s, 3H, *N*CH_3_), 3.91 (t, *J* = 7.36 Hz, 1H, *N*CH), 4.13 (t, *J* = 8.8 Hz, 1H, *N*CH), 4.84 (d, *J* = 6.6 Hz, 1H), 5.78 (dd, *J* = 11.00, 7.32 Hz, 1H), 6.17–7.79 (m, Ar-H, 15H), 8.77 (s, 1H, N-H); ^13^C-NMR (CDCl_3_, 100 MHz): δ 39.8, 40.2, 58.4, 84.4, 109.0, 119.2, 121.0, 123.3, 125.4, 125.5, 126.5, 126.8, 126.9, 127.2, 129.8, 129.9, 130.0, 130.8, 131.0, 131.4, 133.3, 135.3, 138.7, 139.8, 140.9, 142.9, 179.1, 182.6. Mass spectrum (EI, 70 eV): *m*/*z*, 536 (M^+^). Anal. calcd. for C_31_H_23_BrN_2_O_2_: C, 69.54; H, 4.33; N, 5.23; %. Found: C, 69.68; H, 4.48; N, 5.36%.

(2′S*,4′S*)-1′-Methyl-4′-(2-chlorophenyl)-10H-dispiro[anthracene-9,3′-pyrrolidine-2′,3′′-indoline]-2′′,10-dione (**6d**). White solid (270 mg, 87%); ^1^H-NMR (CDCl_3_, 400 MHz): δ 2.20 (s, 3H, *N*CH_3_), 3.97 (t, *J* = 8.8 Hz, 1H, *N*CH), 4.64 (t, *J* = 10.2 Hz, 1H, *N*CH), 5.11 (d, *J* = 7.3 Hz, 1H), 6.14 (dd, *J* = 11.00, 7.32 Hz), 6.32–8.05 (m, Ar-H, 15H); ^13^C-NMR (CDCl_3_, 100 MHz): δ 36.7, 42.1, 58.9, 59.3, 84.2, 108.78, 121.8, 123,6, 125.4, 125.5, 125.8, 126.3, 126.6, 127.1, 127.2, 127.3, 127.4, 127.5, 127.9, 128.1, 128.3, 130.3, 130.5, 130.6, 131.5, 132.1, 132.6, 135.1, 140.6, 179.5, 182.3; Mass spectrum (EI, 70 eV): *m*/*z*, 491 (M^+^). Anal. calcd. for C_31_H_23_ClN_2_O_2_: C, 75.83; H, 4.72; N, 5.71%. Found: C, 75.99; H, 4.81; N, 5.86%.

(2′S*,4′S*)-1′-Methyl-4′-(2,4-dichlorophenyl)-10H-dispiro[anthracene-9,3′-pyrrolidine-2′,3′′-indoline]-2′′,10-dione (**6e**). White solid ( 251 mg, 84%); ^1^H-NMR (CDCl_3_, 400 MHz): δ 2.20 (s, 3H, *N*CH_3_), 3.94 (t, *J* = 8.08 Hz, 1H, *N*CH), 4.61 (t, *J* = 9.5 Hz, 1H, *N*CH), 5.09 (d, *J* = 7.3 Hz, 1H), 6.05 (dd, *J* = 11.00, 7.32 Hz) 6.58–8.09 (m, Ar-H, 14H); ^13^C-NMR (CDCl_3_, 100 MHz): δ 36.2, 41.4, 58.5, 58.6, 84.7, 108.84, 121.4, 123,5, 125.4, 125.5, 125.9, 126.4, 126.5, 127.2, 127.4, 127.4, 127.7, 127.9, 128.3, 128.5, 130.3, 130.8, 132.2, 132.3, 132.6, 135.2, 140.6, 179.8, 183.6; Mass spectrum (EI, 70 eV): *m*/*z*, 526 (M^+^). Anal. calcd. for C_31_H_22_Cl_2_N_2_O_2_: C, 70.86; H, 4.22; N, 5.33%. Found: C, 70.75; H, 4.36; N, 5.48%.

(2′S*,4′S*)-1′-Methyl-4′-(4-chlorophenyl)-10H-dispiro[anthracene-9,3′-pyrrolidine-2′,3′′-indoline]-2′′,10-dione (**6f**). White solid (273 mg, 88%); ^1^H-NMR (CDCl_3_, 400 MHz): δ 2.21 (s, 3H, *N*CH_3_), 4.12 (t, *J* = 8.08 Hz, 1H, *N*CH), 4.32 (t, *J* = 9.52 Hz, 1H, *N*CH), 5.11 (d, *J* = 7.32 Hz, 1H), 5.96 (dd, *J* = 11.00, 7.36 Hz, 1H) 6.36–8.00 (m, Ar-H, 15H); ^13^C-NMR (CDCl_3_, 100 MHz): δ 36.4, 41.7, 58.7, 58.9, 84.7, 108.8, 121.9, 123.4, 125.8, 126.5, 127.1, 127.4, 127.8, 128.1, 128.2, 129.4, 130.3, 130.7, 131.4, 131.5, 132.6 133.4, 135,5, 137.9, 139.6, 140.6, 141.7, 179.0, 182.7. Mass spectrum (EI, 70 eV): *m*/*z*, 491 (M^+^). Anal. calcd. for C_31_H_23_ClN_2_O_2_: C, 75.83; H, 4.72; N, 5.71%. Found: C, 75.73; H, 4.86; N, 5.88%.

(2′S*,4′S*)-1′-Methyl-4′-(2-methylphenyl)-10H-dispiro[anthracene-9,3′-pyrrolidine-2′,3′′-indoline]-2′′,10-dione (**6g**). White solid (254 mg, 80%); ^1^H-NMR (CDCl_3_, 400 MHz): 2.40 (s, 3H, *N*CH_3_), 2.72 (s, 3H, CH_3_), 3.92 (s, 3H, 1H, *N*CH), 4.88 (t, *J* = 8.0 Hz, 1H, *N*CH), 5.06 (d, 1H, *J* = 10.9 Hz), 6.01 (dd, *J* = 11.00, 7.32 Hz, 1H), 6.31–8.25 (m, Ar-H, 15H); ^13^C-NMR (CDCl_3_, 75 MHz): δ 36.9, 42.5, 54.3, 58.2, 59.0, 84.8, 108.7, 120.9, 121.8, 124.9, 125.6, 126.5, 126.7, 127.2, 127.4, 127.5, 127.9, 128.2, 128.3, 128.4, 129.3, 130.2, 131.3, 131.4, 132.7, 133.5, 135,6, 136.4, 140.5, 141,9, 178,6, 183.2. Mass spectrum (EI, 70 eV): *m*/*z*, 472 (M + 1)). Anal. calcd. for C_32_H_26_N_2_O_2_: C, 81.68; H, 5.57; N, 5.95%. Found: C, 81.78; H, 5.69; N, 5.84%.

(2′S*,4′S*)-1′-Methyl-4′-(4-methylphenyl)-10H-dispiro[anthracene-9,3′′-pyrrolidine-2′,3′′-indoline]-2′′,10-dione (**6h**). Colorless crystals (283 mg, 89%); ^1^H-NMR (300 MHz, CDCl_3_) δ_H_: 2.20 (s, 3H, *N*CH_3_), 2.21 (s, 3H, CH_3_), 4,16 (t, *J* = 7.5 Hz, 1H, *N*CH), 4.32 (t, *J* = 8.7 Hz, 1H, *N*CH), 5.11 (d, *J* = 7.5 Hz, 1H), 5.99 (t, *J* = 10.5 Hz, 1H), 6.37–8.0 (m, Ar-H, 15H) ppm; ^13^C-NMR (75 MHz, CDCl_3_) δ_C_: 20.8, 36.4, 41.8, 58.9, 59.0, 84.9, 108.9, 121.8, 123.7, 125.5, 126.6, 126.9, 127.1, 127.5, 127.8, 127.9, 128.7, 130.1, 130.5, 131.5, 132.4, 133.3, 135.0, 135.5, 136.2, 140.1, 141.2, 141.9, 179.3, 182.8. Mass spectrum (EI, 70 eV): *m/z*, 472 (M^+^). Anal. Calcd. For C_32_H_26_N_2_O_2_: C, 81.68; H, 5.57; N, 5.95%. Found: C, 81.76; H, 5.67; N, 5.86%.

(2′S*,4′S*)-1′-Methyl-4′-(3-methoxyphenyl)-10H-dispiro[anthracene-9,3′-pyrrolidine-2′,3′′-indoline]-2′′,10-dione (**6i**). White solid (253 mg, 81%); ^1^H-NMR (CDCl_3_, 400 MHz): 2.21 (s, 3H, *N*CH_3_), 3.49 (s, 3H, OCH_3_), 4.18 (t, *J* = 8.0 Hz, 1H, *N*CH), 4.32 (t, *J* = 10.96 Hz, 1H, *N*CH), 5.11 (d, *J* = 7.32 Hz, 1H), 5.99 (dd, *J* = 11.0, 7.32 Hz, 1H), 6.36–7.99 (m, Ar-H, 15H), 8.43 (s, 1H, NH); ^13^C-NMR (CDCl_3_, 75 MHz): δ 36.5, 42.3, 54.9, 58.7, 59.1, 84.8, 108.8, 110.8, 112.4, 114.3, 120.6, 121.9, 124.1, 125.6, 125.9, 126.7, 127.2, 127.9, 129.0, 131.4, 132.5, 133.5, 135.4, 138.7, 140.0, 141.1, 141.8, 159.2, 179.5, 182.94. Mass spectrum (EI, 70 eV): *m*/*z*, 487 (M^+^). Anal. calcd. For C_32_H_26_N_2_O_3_: C, 78.99; H, 5.39; N, 5.76%; Found: C, 78.87; H, 5.49; N, 5.65%.

(2′S*,4′S*)-1′-Methyl-4′-(4-methoxyphenyl)-10H-dispiro[anthracene-9,3′-pyrrolidine-2′,3′′-indoline]-2′′,10-dione (**6j**). White solid (246 mg, 79%); ^1^H-NMR (CDCl_3_, 400 MHz): 2.20 (s, 3H, *N*CH_3_), 3.65 (s, 3H, OCH_3_), 4.14 (t, *J* = 7.3 Hz, 1H, *N*CH), 4.30 (t, *J* = 8.8 Hz, 1H, *N*CH), 5.10 (d, *J* = 7.3 Hz, 1H), 5.96 (dd, *J* = 11.0, 7.32 Hz, 1H), 6.37–7.99 (m, Ar-H, 15H), 8.8 (s, 1H, N-H); ^13^C-NMR (CDCl_3_, 75 MHz): δ 36.5, 41.4, 55.1, 59.0, 59.1, 85.0, 108.9, 112.5, 113.5, 121.8, 123.7, 124.0, 125.6, 126.7, 127.0, 127.2, 127.8, 127.9, 129.0, 130.2, 130.6, 131.3, 131.6, 132.4, 133.4, 135.5,141.9, 157.4, 179.5, 182.9. Mass spectrum (EI, 70 eV): *m*/*z* 487 (M^+^). Anal. calcd. for C_32_H_26_N_2_O_3_: C, 78.99; H, 5.39; N, 5.76%. Found: C, 78.83; H, 5.46; N, 5.88%.

(2′S*,4′S*)-1′-Methyl-4′-(3-nitrophenyl)-10H-dispiro[anthracene-9,3′-pyrrolidine-2′,3′′-indoline]-2′′,10-dione (**6k**). White solid (236 mg, 77%); ^1^H-NMR (CDCl_3_, 400 MHz): 2.22 (s, 3H, *N*CH_3_), 4.19 (t, *J* = 8.0 Hz, 1H, *N*CH), 4.42 (t, *J* = 10.2 Hz, 1H, *N*CH), 5.10 (d, *J* = 7.3 Hz, 1H), 6.04 (dd, *J* = 11.00, 7.32 Hz, 1H), 6.38–8.02 (m, Ar-H, 16H), 8.98 (s, 1H, N-H); ^13^C-NMR (CDCl_3_, 75 MHz): δ 36.4, 42.3, 58.5, 58.9, 84.7, 109.1, 112.5, 122.0, 126.0, 126.3, 127.4, 127.6, 127.7, 128.3, 128.8, 130.4, 130.8, 131.2, 132.7, 134.3, 135.8, 138.7, 139.1, 140.1, 141.8, 141.9, 148.3, 149.5, 159.6, 179.31, 182.65. Mass spectrum (EI, 70 eV): *m*/*z* 502 (M^+^). Anal. calcd. for C_31_H_23_N_3_O_4_: C, 74.24; H, 4.62; N, 8.38%. Found: C, 74.33; H, 4.55; N, 8.46%.

## 4. Conclusions 

In conclusion, we describe a general, efficient and eco-compatible approach for the regio- and stereoselective synthesis of structurally diverse novel hitherto unexplored dispirooxindole-fused anthrones in excellent yields derived from a one-pot, three component process having a 1,3-dipolar cycloaddition reaction as the key step. These reactions were performed using the ionic liquid, 1-butyl-3-methylimidazolium bromide ([bmim]Br) as the reaction medium.

## Supplementary Materials

Supplementary materials can be accessed at: http://www.mdpi.com/1420-3049/20/09/16142/s1.
