# Supplementary material for: An Expedient Regio- and Diastereoselective Synthesis of Hybrid Frameworks with Embedded Spiro[9,10]dihydroanthracene [9,3′]-pyrrolidine and Spiro[oxindole-3,2′-pyrrolidine] Motifs via an Ionic Liquid-Mediated Multicomponent Reaction
Source: Molecules. 2015 Sep 3;20(9):16142–53. doi: 10.3390/molecules200916142 (PMC6332494; doi:10.3390/molecules200916142)
Supplement: Supplementary file 1 [file molecules-20-16142-s001.pdf]

Supplementary Information

Figure	List of Figures	Page Number
**S1**	^{1}H-NMR spectrum of **6h**	S1
**S2**	Expanded ^{1}H-NMR spectrum of **6h**	S2
**S3**	^{13}C-NMR spectrum of **6h**	S3
**S4**	DEPT spectrum of **6h**	S4
**S5**	^{1}H,^{1}H-COSY spectrum of **6h**	S5
**S6**	^{13}C,^{1}H-COSY spectrum of **6h**	S5
**S7**	Mass spectrum of **6h**	S6

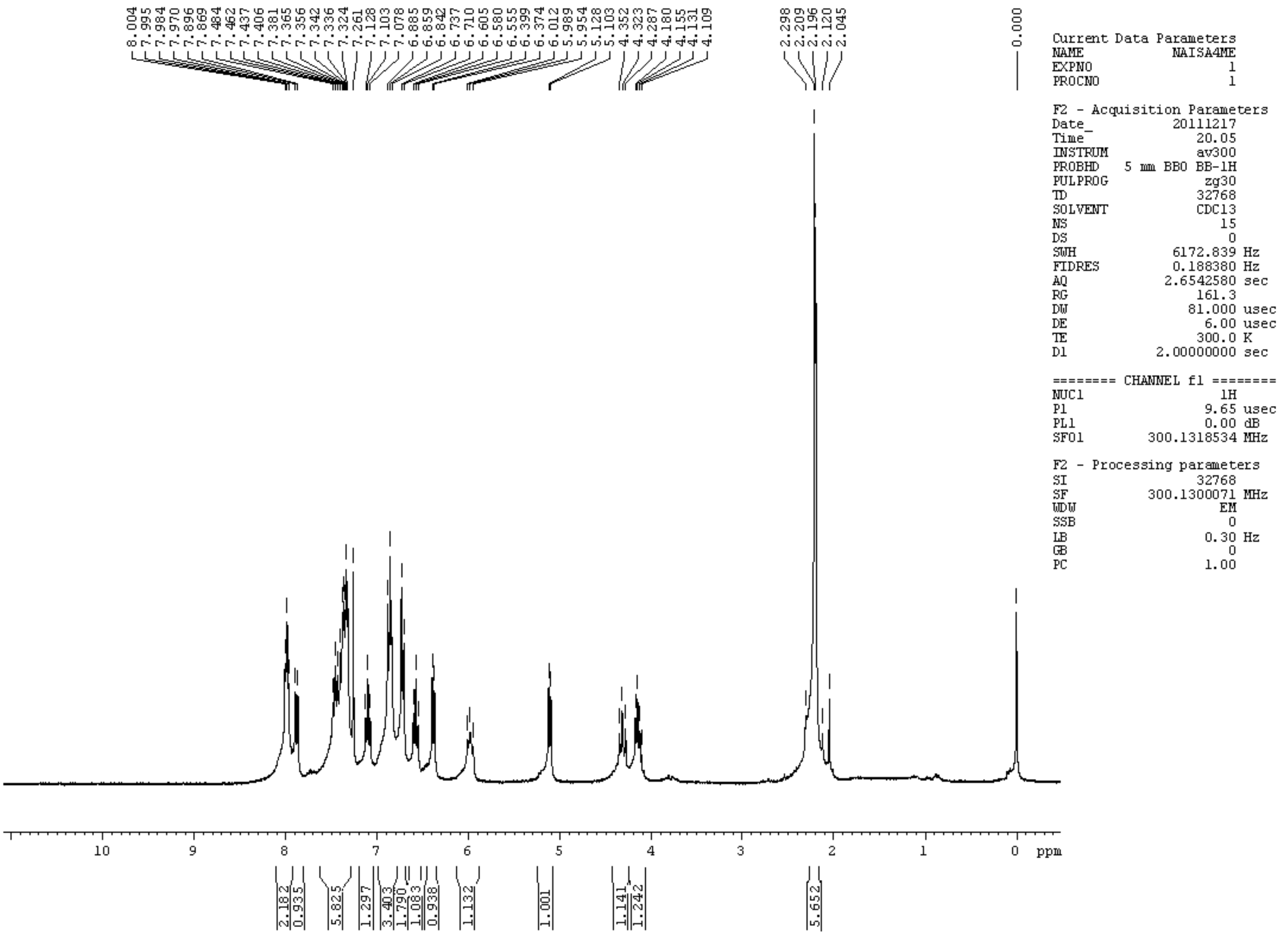

Figure 1. ^{1}H-NMR spectrum of **6h**.

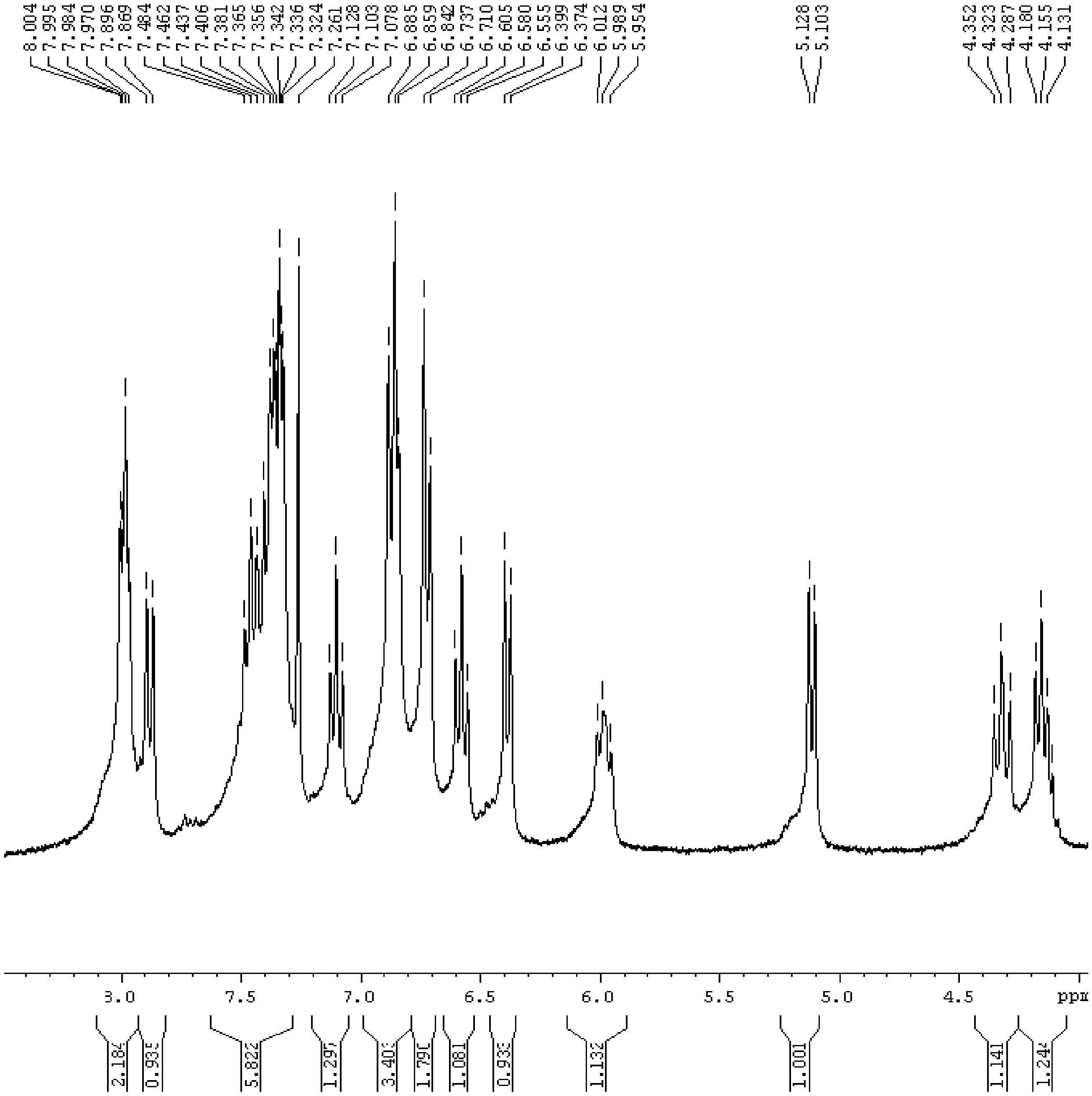

Figure 2. Expanded ^{1}H-NMR spectrum of **6h**.

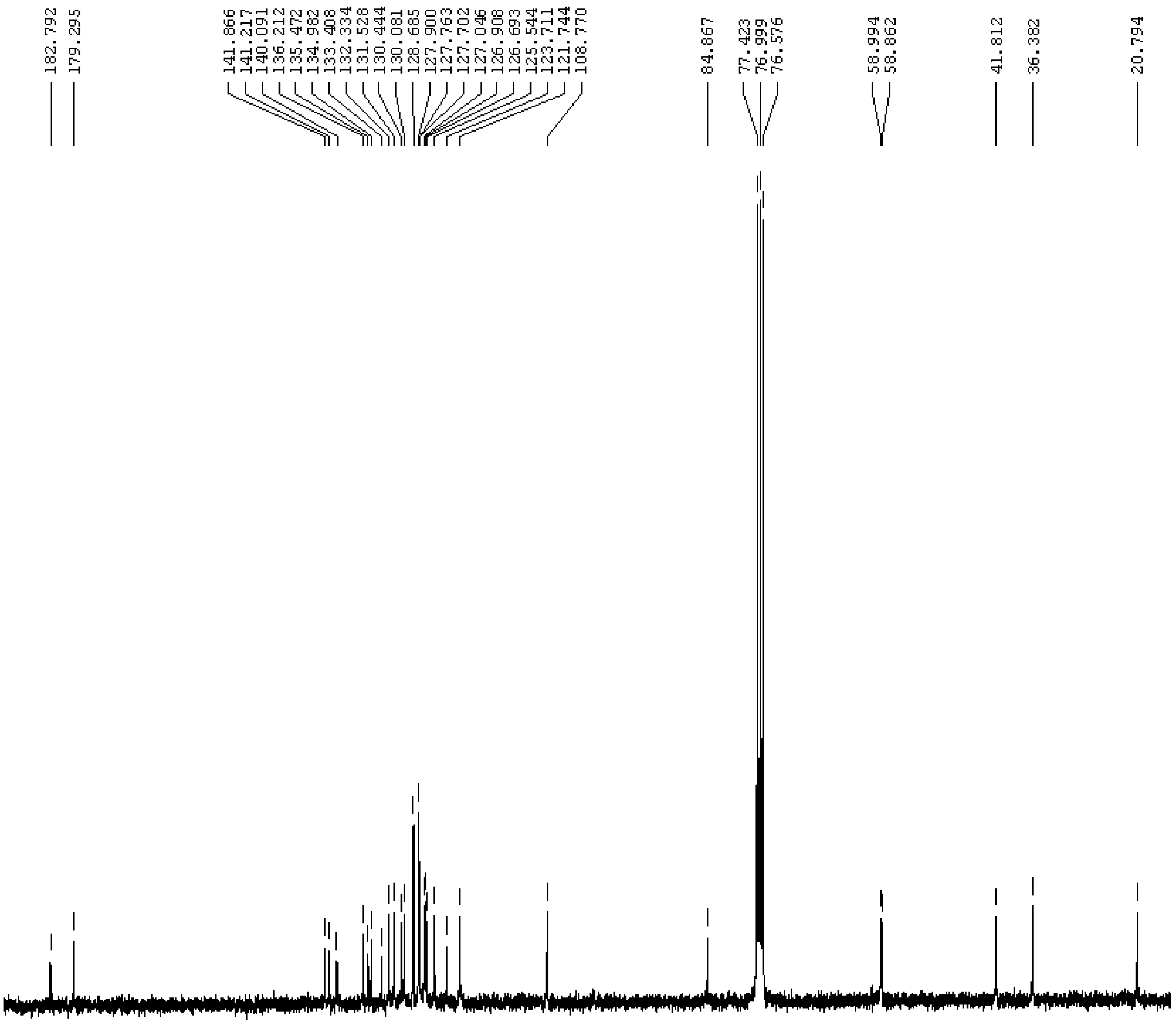

Figure 3. ^{13}C-NMR spectrum of **6h**.

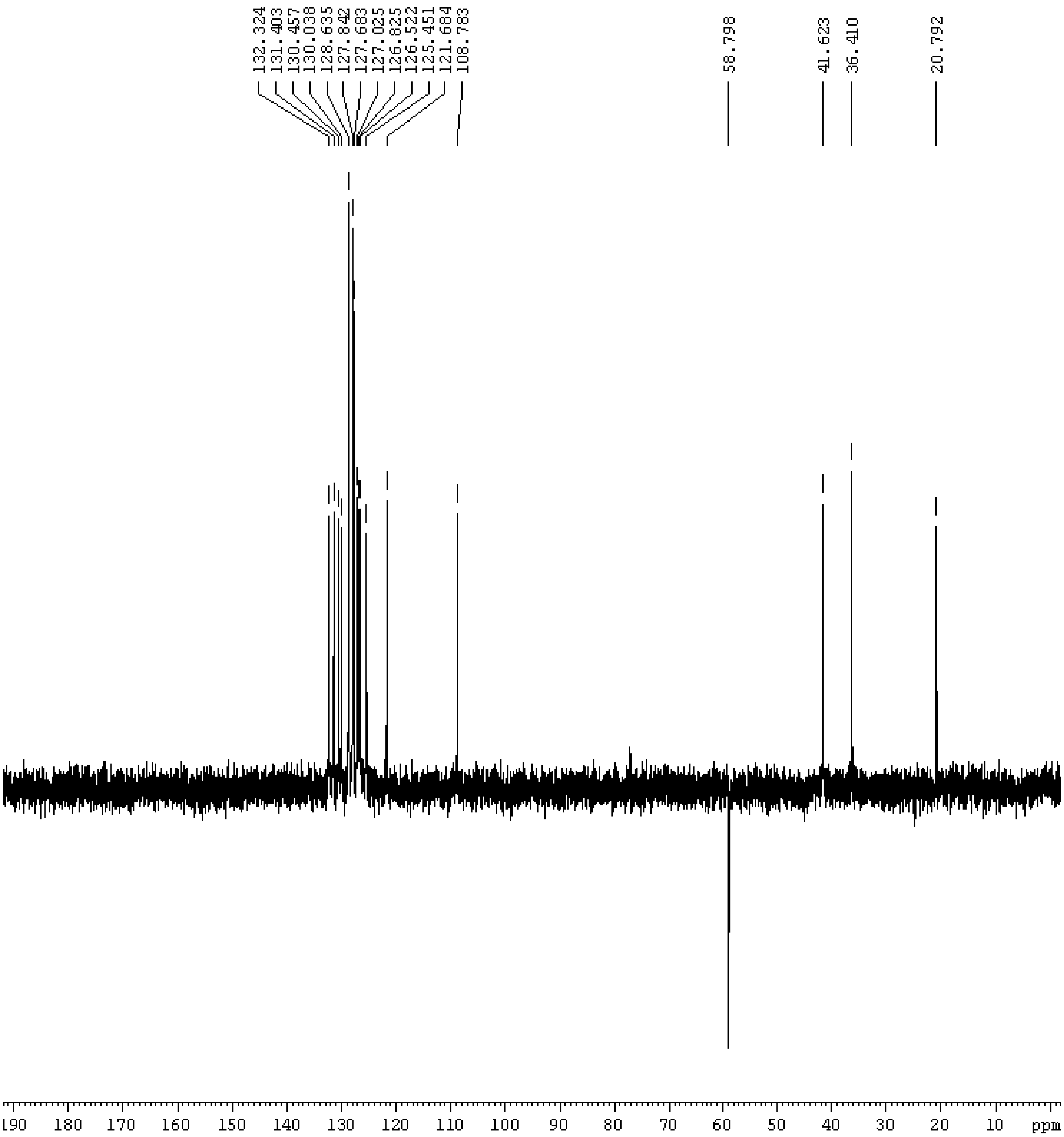

Figure 4. DEPT 135 spectrum of **6h**.

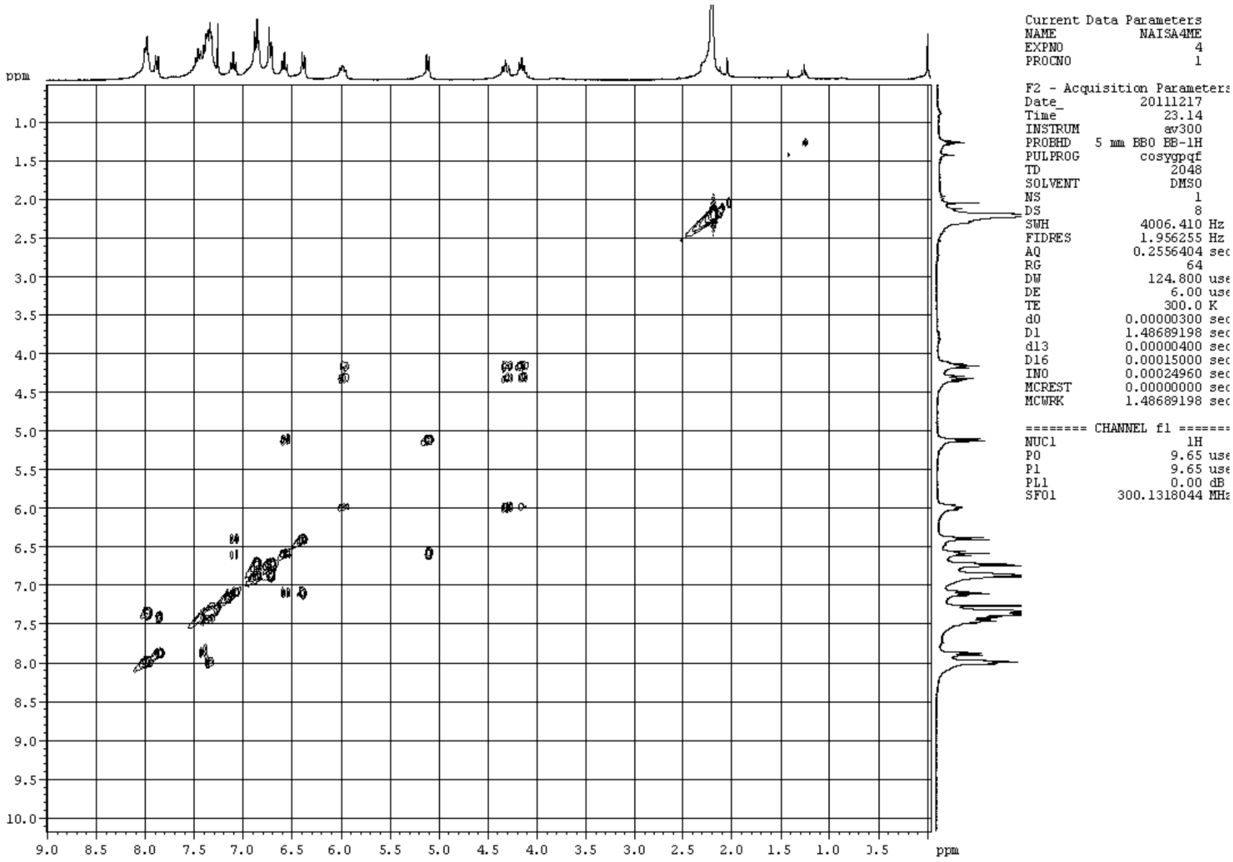

Figure 5. $^{1}H,^{1}H$-COSY spectrum of **6h**.

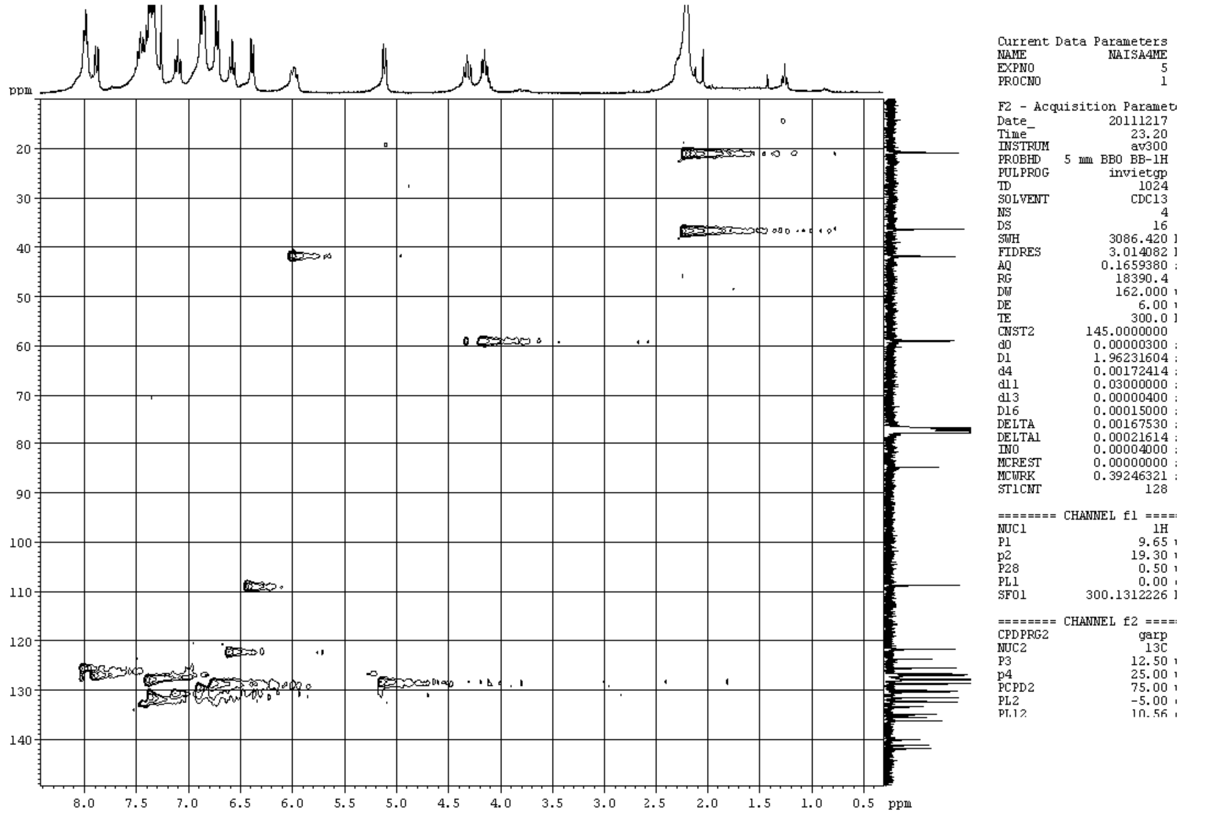

Figure 6. $^{13}C,^{1}H$-COSY spectrum of **6h**.

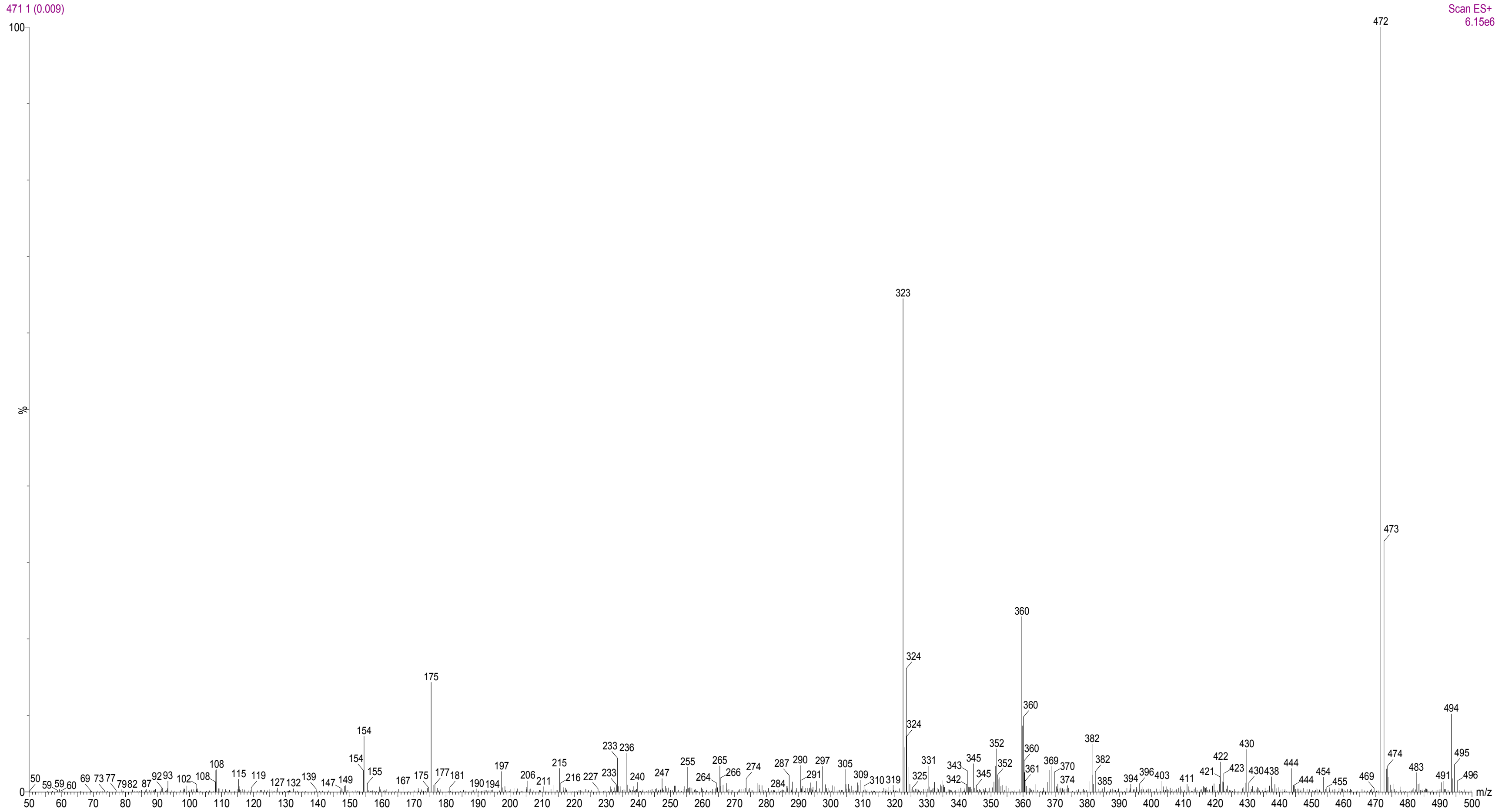

Figure 7. Mass spectrum of **6h**.
